# Sex-specific associations between co-exposure to multiple metals and visuospatial learning in early adolescence

**DOI:** 10.1038/s41398-020-01041-8

**Published:** 2020-10-21

**Authors:** Elza Rechtman, Paul Curtin, Demetrios M. Papazaharias, Stefano Renzetti, Giuseppa Cagna, Marco Peli, Yuri Levin-Schwartz, Donatella Placidi, Donald R. Smith, Roberto G. Lucchini, Robert O. Wright, Megan K. Horton

**Affiliations:** 1 Department of Environmental Medicine and Public Health, Icahn School of Medicine at Mount Sinai, New York, NY USA; 2 Department of Medical and Surgical Specialties, Radiological Sciences, and Public Health, Università degli Studi di Brescia, Brescia, Italy; 3 Department of Clinical Sciences and Community Health, University of Milan, Milan, Italy; 4 Department of Civil, Environmental, Architectural Engineering and Mathematics, Università degli Studi di Brescia, Brescia, Italy; 5 Department of Microbiology and Environmental Toxicology, University of California Santa Cruz, Santa Cruz, CA USA; 6 School of Public Health, Florida International University, Miami, FL USA

**Keywords:** Learning and memory, Diseases

## Abstract

The predisposition, severity, and progression of many diseases differ between males and females. Sex-related differences in susceptibility to neurotoxicant exposures may provide insight into the cause of the observed discrepancy. Early adolescence, a period of substantial structural and functional brain changes, may present a critical window of vulnerability to environmental exposures. This study aimed to examine sex-specific associations between co-exposure to multiple metals and visuospatial memory in early adolescence. Manganese (Mn), lead (Pb), chromium (Cr), and copper (Cu) were measured in blood, urine, hair, nails, and saliva of 188 participants (88 girls; 10–14 years of age). Visuospatial memory skills were assessed using a computerized maze task, the virtual radial arm maze (VRAM). Using generalized weighted quantile sum regression, we investigated sex-specific associations between the combined effect of exposure to the metal mixture and visuospatial working memory and determined the contribution of each component to the outcome. The results suggest that sex moderates the association between the metal mixture and visuospatial learning for all outcomes measured. In girls, exposure was associated with slower visuospatial learning and driven by Mn and Cu. In boys, exposure was associated with faster visuospatial learning, and driven by Cr. These results suggest that (a) the effect of metal co-exposure on learning differs in magnitude, and in the direction between sexes, and (b) early adolescence may be a sensitive developmental period for metal exposure.

## Introduction

Sex-specific differences in the prevalence, severity, and progression of neurodevelopmental diseases is a growing field of interest in biomedical research, motivated by sex-specific behaviors and physiology^1^. Substantial evidence from recent neuroimaging studies support sex differences in human brain structure and function beginning in utero and persisting throughout the life course^2–6^. Longitudinal studies demonstrate robust sexual dimorphism of brain developmental trajectories, specifically during early adolescence^7^. Sex differences in brain development may emerge from endogenous developmental programming, developmental experience, and/or may be shaped through environmental exposures^8,9^. Sex- and gender-related behavioral differences during infancy and childhood have been reported and related to differences in early exposure to chemicals^10^. A more complete understanding of differential vulnerability to neurotoxicants by sex might provide insight into sexual dimorphism of brain, behavior, mental health, and mental disorders^11^.

We focus on adolescence as a sensitive period of brain development^12–14^ and an understudied window of vulnerability to environmental exposures^15,16^. Similar to early life sensitive periods of brain development, the adolescent brain undergoes substantial structural and functional changes involving synaptogenesis, myelination, and synaptic pruning^12,17,18^. Exposure to neuroactive metals (i.e., metals that may have both essential and detrimental impacts) during this period of rapid growth and development may lead to long-lasting changes in neuronal circuitry^19–23^. During adolescence, neural circuitry and synapses are sculpted to support the transition from basic to more complex skills reliant on executive functioning^12^. Executive functions are cognitive processes that enable planning and execution of complex tasks requiring attention, memory, and cognitive flexibility. Working memory, a component of executive functioning, develops gradually throughout childhood and early adolescence^24^. Despite important behavioral, cognitive, and physical developmental changes occurring during this period, the transition to adolescence has received little attention in studies of children’s environmental health. In this paper, we aimed to investigate sex-specific vulnerabilities to the effect of exposure to several metals on visuospatial working memory in adolescents.

Environmental exposures virtually always occur as complex mixtures^25^. Individuals, including children and adolescents, are rarely exposed to one metal at a time and in most cases, are exposed to low levels of several metals simultaneously^26–29^. Co-exposure to multiple metals may influence the toxicity of each metal^30,31^ resulting in adverse effects observed well below the no-observed-adverse-effect-level (NOAEL). Further complicating mixtures research, each mixture subcomponent (i.e., each metal) may follow a different toxicological profile for metabolism and excretion. Exposure biomarkers (urine, blood, saliva, etc.) reflect differences in pharmacokinetics, and therefore, associated health effects may be matrix-dependent^32,33^.

Our study takes place among adolescents residing in Brescia, a heavily industrialized province of northern Italy. We examined the effects of concurrent exposure to a mixture of four neuroactive chemicals: manganese (Mn), lead (Pb), chromium (Cr), and copper (Cu), since they are common in the province of Brescia due to anthropogenic emission from ferroalloy industries. High levels of these metals have been detected in deposited dust samples collected from residential households throughout the province^34^. Mn, Cr, and Cu are essential for several important biological processes in low doses; however, they have been consistently reported as neurotoxic in higher doses^35–37^. Pb is a nonessential, highly neurotoxic heavy metal. To best capture exposure to the overall metal mixture as well as the metal varying metabolic profiles, we used multi-media biomarkers (MMBs)^38^. Concentrations of the four metals were measured in five biological matrices, blood, urine, hair, nails, and saliva. We use a dimension reduction mixtures method—generalized weighted quantile sum (WQS) regression^39–41^—to examine the associations between the mixture and visuospatial working memory. This approach allows for a data-driven selection of metals, and matrices that contribute most to these associations.

The objective of this study was to examine sex-specific vulnerability to the effect of co-exposure to multiple metals on visuospatial working memory during adolescence, a period of considerable developmental and behavioral transition. We examined associations between co-exposure to four metals (Mn, Pb, Cr, and Cu) measured in five matrices (blood, urine, hair, nails, and saliva), and visuospatial learning in early adolescence, and assessed the contribution of each metal to the overall mixture effect.

## Methods

### Participants

The Public Health Impact of Metal Exposure (PHIME) cohort investigates associations between metal exposure from anthropogenic emissions and developmental health outcomes in adolescents and young adults. Details of the study have been described elsewhere^42,43^. Briefly, using a community-based participatory approach, 720 participants aged 10–14 years residing near ferroalloy plants in the Province of Brescia, Italy, were enrolled into PHIME in two phases, 2007 to 2010 (*n* = 312) and 2010 to 2014 (*n* = 408). Inclusion criteria included: birth in the areas of interest, family residence in Brescia for at least two generations, residence in the study areas since birth. Exclusion criteria included: neurological, hepatic, metabolic, endocrine, psychiatric disorders; hand and/or finger deficits; visual deficits not adequately corrected. Among the 408 adolescents who were enrolled in the second study phase, the virtual radial arm maze (VRAM) was administered to 402 participants, and 367 (91%) completed all eight trials as a sub-study designed to promote the use of neurobehavioral instruments that cross animal and human studies (R01ES013744-S). Mn, Pb, Cr, and Cu were measured in blood, urine, hair, fingernails, and saliva of 242, 308, 324, 293, and 329 participants, respectively. Complete exposure data (i.e., all metals in all matrices totaling 20 components), outcome, and covariates data were available for the 188 participants (88 girls) included in this analysis.

This study was approved by the Institutional Review Board of the University of California, Santa Cruz and the Public Health Agency of Brescia. Written informed consent was obtained from all participants.

### Biomarker measures of exposure

Biological samples including venous whole blood, spot urine, saliva, hair, and fingernail clippings were collected from each subject upon enrollment, as described in detail elsewhere^42,44–46^, Supplementary Material 1, and Supplementary Table 1. Biological samples were processed and analyzed for metal concentrations using magnetic sector inductively coupled plasma mass spectrometry (Thermo Element XR ICP-MS), as described elsewhere^42,44–46^.

### VRAM testing procedures

The VRAM, a computerized maze task, was administered to assess visuospatial memory skills^47^. Its administration in our population is described elsewhere^48,49^. Briefly, using a laptop and a joystick, participants navigated a three-dimensional maze comprising eight arms, four of which were baited with rewards. Participants were instructed to retrieve all four rewards as quickly as possible, in a maximum of 180 s. The task was repeated for eight consecutive trials where the same arms remained baited. Participants were instructed to use visual cues in the virtual room to differentiate baited vs. non-baited arms. Performance measures include: (1) the time to complete the maze (seconds), (2) the distance traveled (digital units), (3) the number of working memory errors (i.e., entry into an arm that had already been entered on that trial), and (4) the number of reference memory errors (i.e., entry into an arm that was never baited). For each of these four performance measures, we modeled a learning curve based on the performance across the eight trials.

### Covariate data

Sex was self-reported upon enrollment. IQ was measured using the Wechsler Intelligence Scale for Children 3rd edition (WISC-III)^50^. Socioeconomic status (SES) index (low, medium, high) was calculated according to Cesana et al.^51^, and was composed from parental education and occupation. Due to previous associations between daily frequency of playing video games and VRAM preformance^49^, we included self-reported daily frequency of playing video games.

### Statistical analysis

To test our hypothesis that sex moderates the association between adolescent co-exposure to multiple metals and VRAM performance, we used generalized linear mixed models (GLMMs) and generalized weighted quartile sum (gWQS) regression. Using GLMMs with random slopes, we derived a unique slope for each individual, capturing the change in VRAM performance over time and refer to this slope as the learning curve. Distributions of the time and distance to complete the maze were continuous, working and reference memory errors had count-like distributions. Zero-inflated Poisson mixed models were used to address zero-inflated working and reference memory error distributions. All GLMMs were adjusted for age, SES, daily frequency of playing video games, and IQ^48,49^. GLMMs were implemented in R (v3.5.1) using the lme4 and glmmTMB packages.

We used gWQS to estimate associations between metal mixture exposure and VRAM learning curves. WQS is a mixtures-based ensemble modeling strategy that tests for associations between the combined effect of multiple exposures and an outcome of interest. We included 20 components in the mixture; Mn, Pb, Cr, and Cu measured in blood, urine, hair, nails, and saliva. The WQS analysis is implemented in two steps. First, a weighted index representing the association between each individual metal and the outcome is estimated across 100 bootstrap samples. Second, this weighted index is tested in a linear regression model predicting the association between the mixture and the outcome. Prior to model estimation, all exposures were deciled. The mixture is defined such that WQS = $\Sigma _{i = 1}^cw_iq_{i,j}$ is the sum of the cross products of the empirically estimated weight (*w*_*i*_) for each predictor variable (*i*) and the ranked concentration of that predictor per subject (*q*_*i,j*_). A significance test for the WQS index provides an estimate of the association with the overall mixture, while the weights associated with each predictor provide an indicator of each individual variable’s contribution to the overall effect^39–41^.

To examine the moderating effect of sex on the associations between metal co-exposure and visuospatial learning, we included an interaction term between sex and the metal mixture index; this allows for a direct effect estimate on the multiplicative interaction of these variables and an explicit hypothesis test. To assess the contribution of each metal to the overall mixture effect, we examined associations in each sex independently. We investigated both positive and negative effects of the WQS mixture index on the outcomes. Finally, in a sensitivity analysis, we investigated the possibility of quadratic relation between the mixture and the outcome, by including a quadratic term to the models and assessing the fit of these models using model fit statistics (AIC) and Likelihood-Ratio tests. WQS models were implemented in R (v3.5.1) with the gWQS package. As the learning curves used as outcomes were adjusted for covariates (i.e., in the GLMM), the WQS did not require additional adjustments.

We also examined associations between subsets of metal mixtures within each matrix (i.e., blood Mn, Pb, Cr, and Cu) and each VRAM outcome stratified by sex. Finally, we examined associations between each individual metal in each matrix and VRAM learning curve.

## Results

### Demographics

Sociodemographic characteristics stratified by sex are presented in Table 1. Age and IQ did not differ by sex. The distribution of SES differs between girls and boys (i.e., medium SES = 27% vs. 16%, *p* = 0.02) and boys reported significantly more video game playing than girls (<2 h/day = 21% vs. 12.5%, *p* = 0.01, respectively). In general, participants included in this study (*n* = 188) did not differ from the second phase of the PHIME cohort (*n* = 408) with respect to the covariates reported in Table 1 (Supplementary Table 2).

**Table 1:** Sex-stratified sociodemographic characteristics of adolescents enrolled in the PHIME cohort who were selected into the current study (*n* = 188).

Characteristic	Girls (*n* = 88) Mean ± SD or %	Boys (*n* = 100) Mean ± SD or %	p^*^
Age (years)	12.1 ± 0.9	12.1 ± 0.8	0.51
SES			
Low	46.6%	62%	Ref
Medium	27.3%	16%	0.02*
High	26.1%	22%	0.16
Daily frequency of playing video games			
Do not play	12.5%	2%	Ref
Rarely play	26.1%	20%	0.06
<1 h/day	44.3%	50%	0.01*
<2 h/day	12.5%	21%	0.01*
<3 h/day	3.4%	6%	0.03*
>3 h/day	1.1%	1%	0.38
IQ	107.4 ± 13.6	107.1 ± 12.6	0.86

Differences between boys and girls in the distribution of variables were tested using logistic regression.

*SES* socioeconomic status, *IQ* intelligence quotient, measured using the Wechsler Intelligence Scale for Children, 3rd edition (WISC-III).

*p < 0.05.

Metal concentrations in different matrices are reported in Table 2. Overall, the distributions of metal concentration did not differ between boys and girls. However, blood Pb and hair Mn were higher in girls compared to boys (*p* < 0.05, *p* < 0.01, respectively). Hair Cu concentrations were higher among boys (*p* < 0.01).

**Table 2:** Metal concentrations (Mn, Pb, Cr, and Cu) measured in blood, urine, hair, nails, and saliva collected from 188 adolescents from the PHIME cohort included in the current study.

Metal concentration	All participants GM ± GSD	Girls (*n* = 88) GM ± GSD	Boys (*n* = 100) GM ± GSD	*p*^a^
Blood (µg/L)				
Mn	10.92 ± 1.36	10.98 ± 1.39	10.87 ± 1.34	0.68
Pb	14.48 ± 1.80	12.53 ± 1.71	16.45 ± 1.82	0.10
Cr	0.74 ± 2.04	0.69 ± 2.01	0.78 ± 2.06	0.66
Cu	845.17 ± 1.16	827.88 ± 1.16	860.68 ± 1.15	0.02*
Urine (µg/L)^b^				
Mn	0.23 ± 3.26	0.25 ± 3.25	0.20 ± 3.26	0.98
Pb	0.69 ± 1.81	0.67 ± 1.82	0.72 ± 1.80	0.84
Cr	0.19 ± 1.86	0.18 ± 1.77	0.19 ± 1.93	0.28
Cu	8.46 ± 1.68	8.28 ± 1.61	8.63 ± 1.75	0.60
Hair (µg/g)				
Mn	0.07 ± 2.17	0.05 ± 1.97	0.09 ± 2.22	0.004**
Pb	0.10 ± 3.54	0.08 ± 3.47	0.11 ± 3.55	0.17
Cr	0.04 ± 2.12	0.04 ± 2.06	0.04 ± 2.17	0.69
Cu	12.16 ± 1.70	13.73 ± 1.73	10.93 ± 1.64	0.005**
Nails (µg/g)				
Mn	0.27 ± 3.81	0.26 ± 3.74	0.27 ± 3.91	0.57
Pb	0.24 ± 4.54	0.22 ± 4.89	0.25 ± 4.26	0.36
Cr	0.17 ± 2.33	0.19 ± 2.29	0.15 ± 2.35	0.38
Cu	2.51 ± 1.73	2.45 ± 1.84	2.56 ± 1.63	0.99
Saliva (µg/L)				
Mn	3.26 ± 2.95	2.97 ± 2.98	3.54 ± 2.92	0.64
Pb	0.32 ± 3.84	0.30 ± 3.44	0.35 ± 4.22	0.23
Cr	0.30 ± 2.60	0.29 ± 2.33	0.30 ± 2.85	0.46
Cu	16.63 ± 2.90	18.91 ± 2.76	14.85 ± 2.99	0.38

*GM* geometric mean, *GSD* geometric standard deviation.

*p < 0.05, **p < 0.01.

^a^Differences between boys and girls in the distribution of variables were tested using linear regression adjusted for all covariates from Table 1.

^b^Normalized to creatinine concentrations.

### Sex-specific effects of exposure to metal mixtures and visuospatial memory

**Fig. 1:**
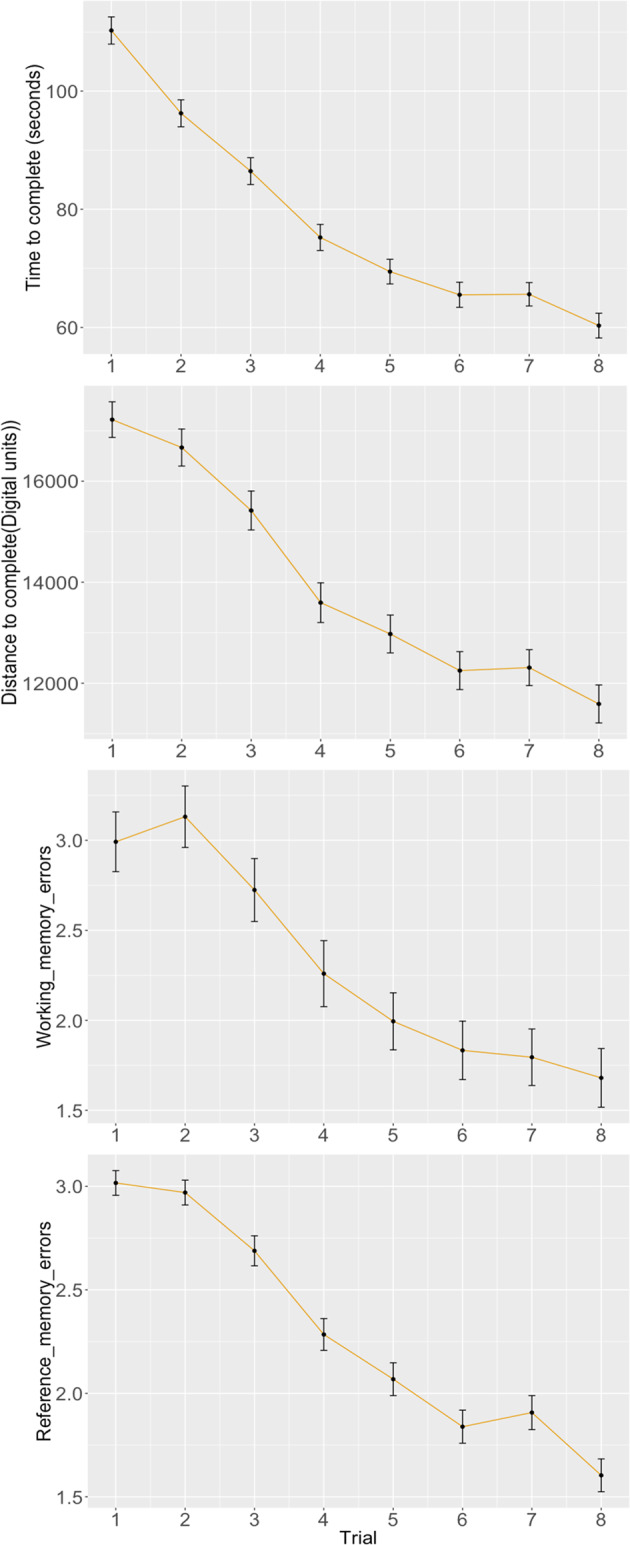
VRAM performance across eight trials. All VRAM outcomes were significantly associated with the trial number, with each consecutive trial lowering the time to complete the task by 6.7 s/trial (95% CI −7.4, −6.4), the distance to complete the task by 844 digital units/trial (95% CI −960, −728), the number of working memory errors by 0.17 errors/trial (95% CI −0.19, −0.14), and the number of reference memory errors by 0.14 errors/trial (95% CI −0.15, −0.12). Dots represent mean performance at each trial and error bars represent standard errors.

Learning curves (shown in Fig. 1) show that, in general, performance on all VRAM measures improved throughout the eight trials. All VRAM outcomes were significantly associated with the trial number, with each consecutive trial lowering the time to complete the task by 6.7 s/trial (95% CI −7.4, −6.4), the distance to complete the task by 844 digital units/trial (95% CI −960, −728), the number of working memory errors by 0.17 errors/trial (95% CI −0.19, −0.14), and the number of reference memory errors by 0.14 errors/trial (95% CI − 0.15, −0.12).

**Fig. 2:**
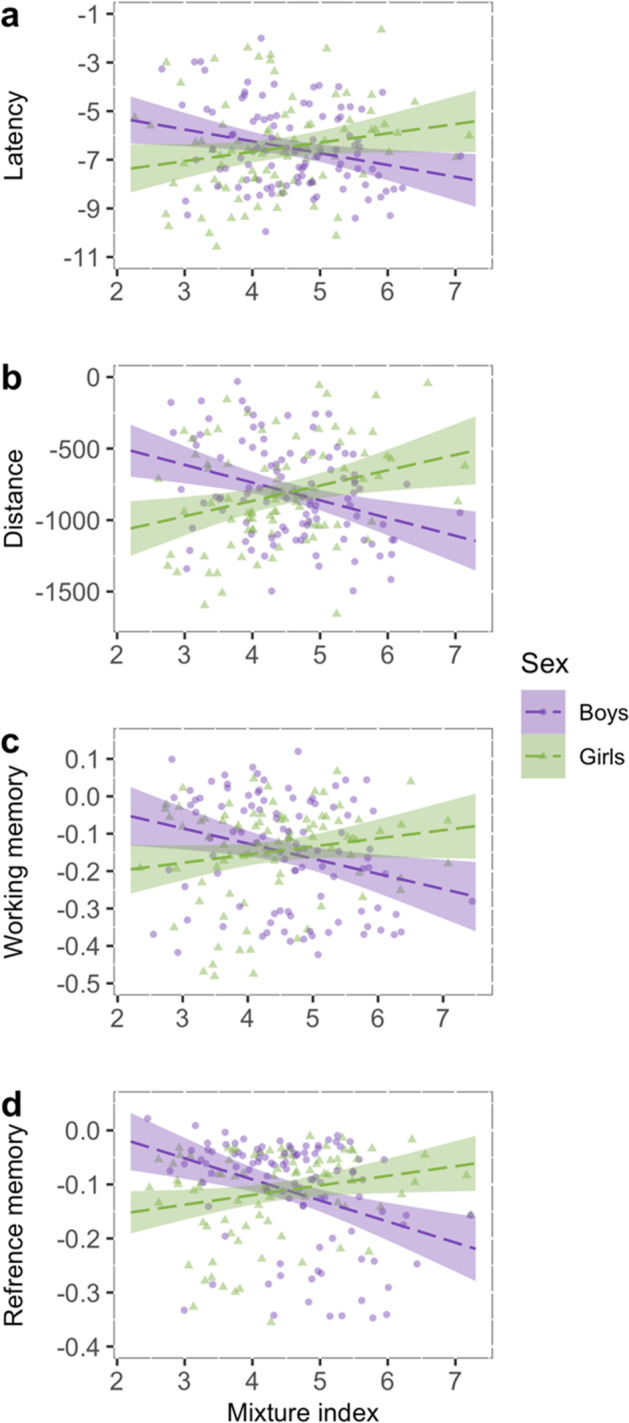
The moderating effect of sex on the association between the WQS metal mixture index and VRAM learning curve among 188 adolescents included in the current study. Results from WQS analyses of learning curves in four VRAM outcomes: **a** time to complete by trial, **b** distance by trial, **c** number of working memory errors by trial, and **d** number of reference memory errors by trial. Female regression lines and SE are color coded in green and male regression lines and SE are color coded in purple.

**Fig. 3:**
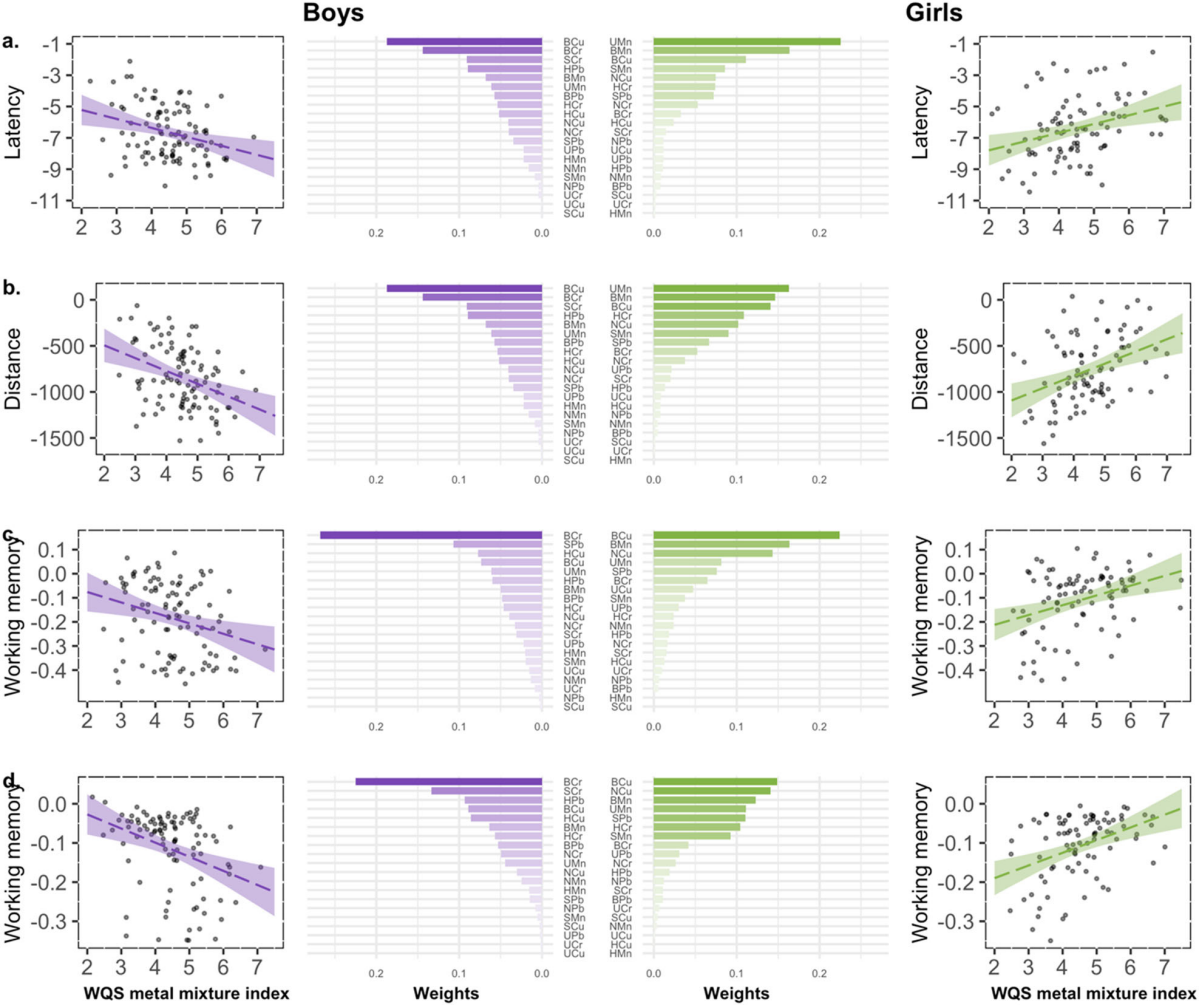
Sex-stratified associations between the WQS metal mixture index and VRAM learning curves among 188 adolescents included in the current study. Results from WQS regressions of learning curves in four VRAM outcomes stratified by sex: **a** latency, **b** distance, **c** number of working memory errors, and **d** number of reference memory errors. Girls regression lines and weights are color coded in green and boys regression lines and weights are color coded in purple. Bar plots show estimated weights for each component of the mixture in the WQS regressions. Weights are gradient color coded to indicate contributions to the overall mixture effect. Components abbreviations: first letter represents the matrix (B = blood, U = urine, H = hair, N = nails, S = saliva), and the second and third letters represent the metal (Mn = manganese, Pb = lead, Cr = chromium, Cu = copper). Only significant models are shown.

Results from gWQS analysis suggest sex-specific vulnerabilities to the combined effect of metal exposure on visuospatial learning in adolescents (i.e., higher metal concentrations were differentially associated with visuospatial learning in girls compared to boys) in all VRAM outcomes: time to complete the maze (*β* = 0.87, *p* < 0.01), the distance traveled (*β* = 222, *p* < 0.001), the number of working memory errors (*β* = 0.06, *p* < 0.01), and the number of reference memory errors (*β* = 0.06, *p* < 0.001) (Fig. 2, Supplementary Table 3). In sex-stratified models, we observed a positive association between the metal mixture index and visuospatial learning in girls (i.e., higher metal concentrations were associated with slower visuospatial learning), and a negative association between the metal mixture index and visuospatial learning in boys (i.e., higher metal concentrations were associated with faster visuospatial learning) (Fig. 3, Supplementary Table 4). In girls, Mn and Cu had the largest weights, with Mn (in all matrices combined) contributing between 31% and 49% of the weights, and Cu (in all matrices combined) contributing between 22% and 43%, depending on the VRAM outcome. In boys, Cr had the largest weight, with its contribution (in all matrices combined) ranging between 33% and 47%, depending on the VRAM outcome. See specific metal weights in each matrix in Supplementary Table 5.

We did not observe significant associations between exposure to any sub-mixture (i.e., all metals in one matrix or a single metal in all matrixes) and VRAM learning curves after correction for multiple comparisons (Supplementary Fig. 1). In addition, no significant associations were observed between individual metals measured in each matrix and VRAM outcomes (data not shown). Additionally, WQS model fit with a quadratic term was not significantly different from model fit without a quadratic term. Finally, WQS models with an interaction term with sex fitted the data significantly better than models that did not include an interaction term (Supplementary Table 6).

## Discussion

In this study of young adolescents, the effect of co-exposure to a mixture of neuroactive metals on VRAM performance differed by sex suggesting sex-specific vulnerability to metal exposure on visuospatial learning. In girls, exposure to the metal mixture was associated with slower visuospatial learning and was predominantly driven by Mn and Cu. In boys, exposure was associated with faster visuospatial learning, and was predominantly driven by Cr. These results suggest that (a) the effect of co-exposure to neuroactive metals on learning differs by sex and (b) early adolescence may be a sensitive developmental period for metal exposure.

Despite well-established associations between metal exposure and neurologic outcomes throughout the life course (refs. ^22,52–56^), only recent investigations have explored sex-specific effects of metals on neurodevelopment^52^. Within PHIME, Bauer and colleagues reported a negative effect of prenatal exposure to Mn measured in deciduous teeth and VRAM outcomes in girls but not in boys^48^. Other studies have also reported associations between higher metal levels and worse cognitive and behavioral outcomes in girls^53–57^. Metal-related neurodevelopmental outcomes have been also reported in boys^58–63^. Our results correspond with previous studies, suggesting that male and female neurodevelopment is not equally vulnerable to the effect of metal exposure. Moreover, our results emphasize that the effect of neurotoxicants does not merely differ in magnitude between the sexes, but also in the direction of the effect. In order to interpret the direction of the association between the metal mixture index and the outcome, the contribution of each metal to the overall mixture effect should be considered. Indeed, a clear distinction between the contribution of the different metals to the overall association is noticeable in boys compared to girls. The positive effect of metals exposure on visuospatial learning in boys is driven by Cr which may be a consequence of its essential role in sugar and fat metabolism^64^.

Our findings suggest an impact of metal exposure on learning during early adolescence. Sex-specific associations between metal exposure and visuospatial learning during early adolescence may arise from dimorphic brain development, mediated by sex chromosomes, gonadal steroid hormones, and other epigenetic factors^65^. Metal exposure during adolescence may affect pubertal sex-dependent brain maturational processes related to visuospatial skills. Sex differences in adolescent behavior and brain maturation have been consistently reported^66,67^. For example, total cerebral volume peaks at age 10.5 years in females and 14.5 years in males. Likewise, gray matter trajectories peak 1–2 years earlier in females^7,68,69^. Working memory abilities improve significantly throughout adolescence with increased accuracy and decreased reaction times as a function of age^70–72^. Neuroimaging studies have confirmed that the neural substrates supporting working memory mature later with gray and white matter changes occurring well into adulthood^73–76^. For example, increased neural specialization of working memory networks during adolescence have been reported, with a shift from early long‐range connections to a later mature local circuitry^77^. Finally, interactions between sex and age-related brain maturation of regions involved in working memory have been reported during adolescence^78,79^.

Our findings demonstrate effects of metal exposure when investigated as a mixture, but not when each metal was investigated individually. This highlights the importance of investigating co-exposure to metals over the traditional approach of investigating one metal at a time. By examining these metals as a mixture and including multiple biological matrices, we capture additional information regarding their varying toxicological profiles. Indeed, performing individual analyses could have led to the false conclusion of null results, suggesting the existence of safe levels of exposure to metals. The National Institute of Environmental Health Sciences (NIEHS) has recognized the importance of combined exposure studies by declaring it a research priority^80^, yet guidelines are still established based on single metal studies which may fail to protect the developing brain. Our findings, if replicated in different populations, may guide appropriate human risk assessments of metal exposure based on mixture approaches.

We selected an animal–human analog task to investigating the effect of complex mixtures on cognition to address our long-term goal of coordinating our findings with animal studies and promote translational research. Animal models' studies are a cornerstone of human toxicology safety evaluation as they are cost-effective, have fast development, and are relatively easy to implement^81,82^. Animal research has the additional advantage of access to neurological tissue enabling mechanistic research. A major challenge for translating results between epidemiology studies and animal studies is the comparability of behavioral outcomes^49^. Our results show that the VRAM task is effective for detecting metal-related visuospatial memory deficits in humans^83,84^. If such findings are replicated in animal models, we propose that mechanistic data from such a study would be more relevant and translatable to humans.

This study has several limitations. First, our small sample size prohibited splitting the sample into training and validation subsets, limiting the generalization of our findings to other populations. Second, biological samples were collected concurrent with the outcomes, thus represent only recent metal exposure. Third, sex differences in unmeasured covariates as well as in possible unmeasured exposures may bias our results. Finally, modeling the learning curves as linear may mask variability in the shape of individual learning curves. These findings should be confirmed in a different and larger population with longitudinal exposure history and outcomes assessment.

The main strength of our study over previous studies investigating effects of metals on neurodevelopment is that we considered sex as an effect modifier rather than a simple covariate. This approach allowed us to detect sex-specific effects which may inform the implementation of sex-specific protections and regulations. In addition, the MMBs^38^ approach has proven to provide additional information about metals neurotoxicity compared to single matrix investigation and may inform matrix selection in future research. Finally, our choice of a human–animal analog task as an objective measure of visuospatial learning should enable translational research to better address underlying neurotoxic mechanisms and guide additional studies in future research.

## Conclusions

Exposure to metals during early adolescence may have a sex-specific impact on visuospatial learning. These results suggest that (a) the effect of metal co-exposure on learning differs in magnitude, and in the direction between the sexes, and (b) early adolescence may be a sensitive developmental period for metal exposure.

## Supplementary information

SUPPLEMENTAL MATERIAL
