# Supplementary material for: Sex-specific associations between co-exposure to multiple metals and visuospatial learning in early adolescence
Source: Transl Psychiatry. 2020 Oct 21;10:358. doi: 10.1038/s41398-020-01041-8 (PMC7578810; doi:10.1038/s41398-020-01041-8)
Supplement: Supplementary file 1 — SUPPLEMENTAL MATERIAL [file 41398_2020_1041_MOESM1_ESM.docx]

Supplementary material

**Supplementary Material 1.** Biomarker collection, analysis protocols and analytical detection limits.

Exposure biomarker samples (blood, urine, hair, fingernails, saliva) were collected from each subject, as described in detail elsewhere. (Smith et al., 2007; Eastman et al., 2013; Lucas et al., 2015; Butler et al., 2018) Briefly, whole blood samples were collected using a 19-gauge butterfly catheter into Li-heparin Sarstedt Monovette Vacutainers. A spot urine sample was collected into a collection cup, and an aliquot removed for creatinine measurement. Passive unstimulated saliva samples were collected directly into trace metal clean microfuge tubes via a 5 cm plastic straw; prior to collection, subjects rinsed their mouths three-times with ultrapure Milli-Q water, then waited 10 min before dispensing saliva into the tube. Whole blood, urine, and saliva samples were stored frozen (-20 °C) until processing for analysis. Hair samples (2-3 cm section of hair from the occipital lobe, proximal to the scalp) were collected using stainless steel scissors. Fingernail samples were collected using stainless steel nail clippers. Hair and nail samples were stored at room temperature until processed for analysis. In addition, physiological iron status was assessed in the children via measures of blood hemoglobin, total serum iron, and serum ferritin and transferrin levels; for all iron status outcomes, the levels were within the normal clinical range (Lucchini et al., 2012).

Biological samples were processed and analyzed for metal concentrations as follows (Lucchini et al., 2012; Eastman et al., 2013; Smith et al., 2007; Lucas et al., 2015; Butler et al., 2018): For whole blood, samples were thawed and ~0.25 mL of blood was mixed with 0.5 mL 15.7 N quartz distilled nitric acid in a polyethylene tube, and the mixture left overnight at room temperature. Subsequently, 0.25 mL of Ultrex 30% hydrogen peroxide was added, followed by 4 mL of Milli-Q water. The mixture was vortexed and the precipitate allowed to settle overnight. Subsequently, 0.5 mL of supernatant was removed and centrifuged at 13000 x g for 10 minutes for analysis. Urine samples were thawed and acidified (pH<2) with 15.7 N quartz distilled nitric acid, and an aliquot removed and centrifuged (13000 x g for 10 minutes) for analysis. Saliva samples were vortexed and centrifuged at 1000 x g for 1 minute, and a 0.1 mL aliquot removed and mixed with 0.4 mL 0.8 N quartz distilled nitric acid, and left overnight. Subsequently, the saliva samples were vortexed and centrifuged (13000 x g for 10 minutes) for analyses.

Hair and fingernail samples were cleaned of exogenous metal contamination as described in Eastman et al. (2012). Briefly, samples were placed in 5 mL syringe bodies (hair) or 1.5 mL microfuge tubes (nails) and sonicated (20 min) in 0.5% Triton, rinsed five-times with ultrapure Milli-Q water, sonicated (10 min) in 1 N trace metal grade nitric acid, rinsed with 1 N nitric acid, and rinsed five-times with Milli-Q water. Clean hair and nail samples were dried at 65 ºC for 48 hours in a HEPA filtered-air clean room. Subsequently, hair samples were digested in 0.5 mL 15.7 N quartz-distilled nitric acid at 80 ºC for 6 h in a Class-100 HEPA filtered-air fume hood. After complete digestion of the hair, samples were diluted with 5 mL Milli-Q water. For analyses, 0.25 mL was transferred to microfuge tube, diluted with 0.25 mL Milli-Q water, and centrifuged at 13000 x g for analysis. Nail samples were digested in 0.1 mL 15.7 N quartz distilled nitric acid at 80 ºC for 4 h in a Class-100 HEPA filtered-air hood. After complete digestion, 1.2 mL Milli-Q water was added and samples centrifuged at 13000 x g for 10 minutes prior to analyses. Rhodium and thallium were added to all samples as internal standards. Metals concentrations (Mn, Pb, Cr, Cu) in all biomarkers were measured using magnetic sector inductively coupled plasma mass spectrometry (Thermo Element XR ICP-MS), as described elsewhere (Smith et al., 2007; Eastman et al., 2013; Lucas et al., 2015; Butler et al., 2018).

**Supplementary Table 1.** Analytical detection limits for metals.

| **Biomarkers** (ng/mL, as analyzed) | Mn | Pb | Cr | Cu |
| --- | --- | --- | --- | --- |
| Blood | 0.018 | 0.015 | 0.002 | 0.061 |
| Hair | 0.006 | 0.002 | 0.003 | 0.066 |
| Nails | 0.033 | 0.017 | 0.012 | 0.044 |
| Saliva | 0.011 | 0.004 | 0.009 | 0.032 |
| Urine | 0.004 | 0.003 | 0.003 | 0.011 |

**Supplementary Table 2.** Sociodemographic characteristics of study participants included between 2010 and 2014 in the parent PHIME cohort and those included in the current analyses.

Note. SES socioeconomic status, IQ = intelligence quotient.

^a^ Missing data for covariates among subjects in the whole cohort; sex (n = 2), age (n = 2), SES (n = 19), daily frequency of playing video games (n = 15), IQ (n = 4).

^b^ Differences between the whole cohort and the current analyses in the distribution of variables were tested using Fisher's Exact tests for of categorical variables and using t tests for continuous variables.

**Supplementary Table 3.** The moderating effect of sex on the association between the WQS metal mixture index and VRAM performance among 188 adolescents included in the current study

| VRAM outcome | β_WQS * Sex_ | SE | p value | p value (corrected)^a^ |
| --- | --- | --- | --- | --- |
| Time to complete  (seconds/trial) | 0.87 | 0.28 | 0.00251 | **0.0048**** |
| Distance to complete  (digital units/trial) | 221.61 | 53.41 | 0.00005 | **0.0001***** |
| Working memory  (errors/trial) | 0.06 | 0.02 | 0.00241 | **0.0048**** |
| Reference memory  (errors/trial) | 0.06 | 0.01 | 0.00004 | **0.0001***** |

β_WQS * Sex_= weighted quartile sum regression interaction coefficient; SE = standard error. Models were not adjusted for covariates since participants individual learning curve was already adjusted from previous analyses.

^a^ p- values were false discovery rate (FDR) corrected for multiple comparisons.

** p < 0.01

*** p < 0.005

**Supplementary Table 4.** Association between the WQS metal mixture index and VRAM learning curves by sex (girls/boys) among 188 adolescents included in the current study.

WQS_β_ = weighted quartile sum regression coefficient; SE = standard error. Models were not adjusted for covariates since participants individual learning curve was already adjusted from previous analyses.

^a^ p- values were false discovery rate (FDR) corrected for multiple comparisons.

* p < 0.05

** p < 0.01

**Supplementary Table 5.** Detailed weights from the gWQS analyses.

- 1.
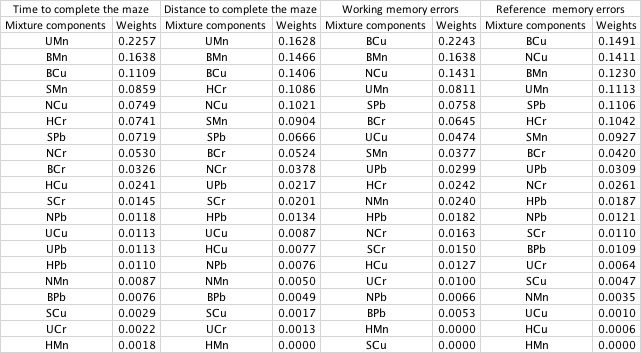
Girls
  2.
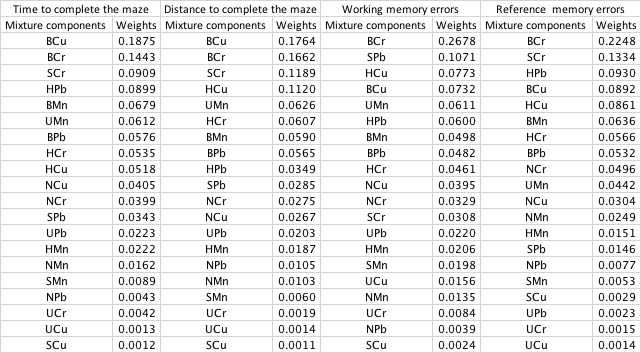
Boys

**Supplementary Table 6.** Likelihood-Ratio test comparing WQS models with and without the interaction term with sex.

| **VRAM outcome** | **Tested models** | **Chi-squared** | **p** |
| --- | --- | --- | --- |
| **Latency** | ~ wqs + Sex + wqs*Sex | 7.25 | 0.0071 |
|  | ~ wqs + Sex |  |  |
| **Distance** | ~ wqs + Sex + wqs*Sex | 13.24 | 0.0002 |
|  | ~ wqs + Sex |  |  |
| **Working Memory Errors** | ~ wqs + Sex + wqs*Sex | 5.81 | 0.0159 |
|  | ~ wqs + Sex |  |  |
| **Reference Memory Errors** | ~ wqs + Sex + wqs*Sex | 13.59 | 0.0002 |
|  | ~ wqs + Sex |  |  |

**Supplementary
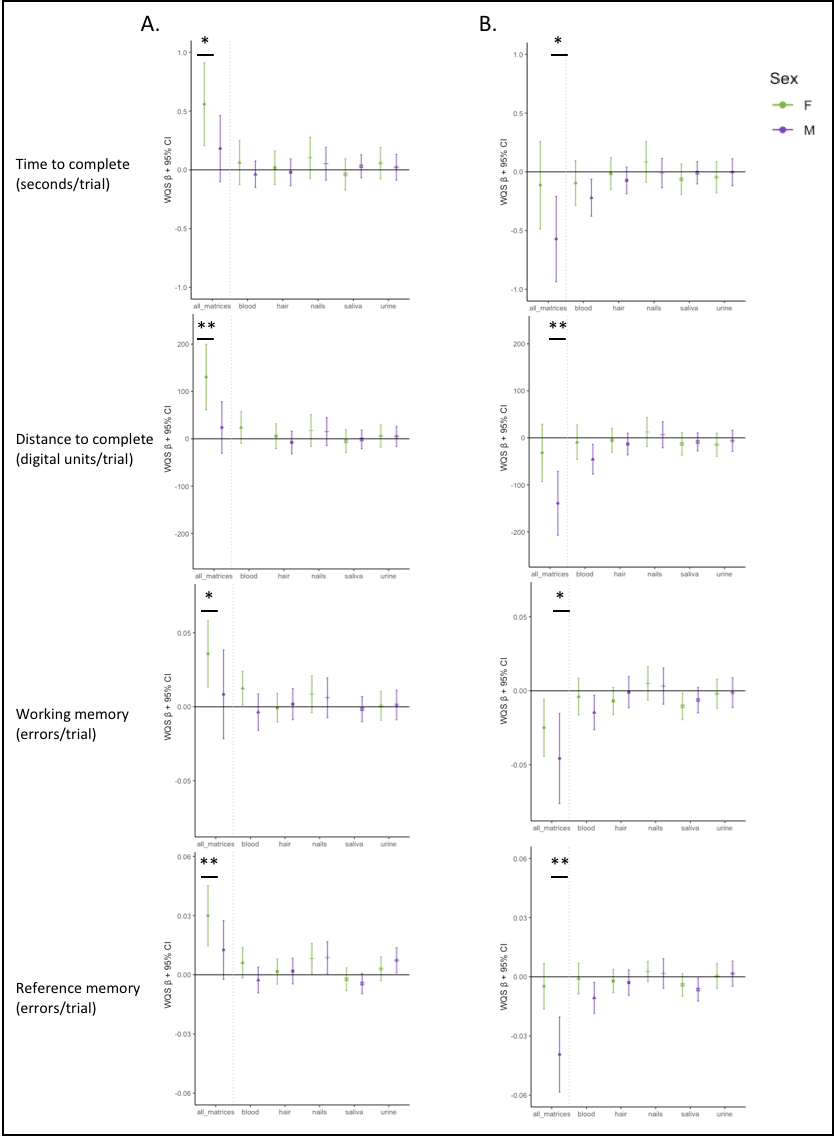
Figure 1.** Associations between all mixtures and sub-mixtures and VRAM performance by sex and by effect direction: A. positive association. B. Negative association.

Beta coefficients and 95% confidence intervals of the association between each matrix and and each VRAM learning curves. * p < 0.05, ** p < 0.01
